# Gut Metabolites Acting on the Gut-Brain Axis: Regulating the Functional State of Microglia

**DOI:** 10.14336/AD.2023.0727

**Published:** 2024-04-01

**Authors:** Wenze Deng, Pengcheng Yi, Yanhong Xiong, Jun Ying, Yue Lin, Yao Dong, Gen Wei, Xifeng Wang, Fuzhou Hua

**Affiliations:** ^1^Department of Anesthesiology, the Second Affiliated Hospital of Nanchang University, Nanchang, Jiangxi, China.; ^2^Key Laboratory of Anesthesiology of Jiangxi Province, Nanchang City, Jiangxi, China.; ^3^Department of Anesthesiology, the First Affiliated Hospital of Nanchang University, Nanchang, Jiangxi, China.

**Keywords:** gut metabolites, microglia, neuroinflammation, gut-brain axis, neurodevelopmental disorders

## Abstract

The gut-brain axis is a communication channel that mediates a complex interplay of intestinal flora with the neural, endocrine, and immune systems, linking gut and brain functions. Gut metabolites, a group of small molecules produced or consumed by biochemical processes in the gut, are involved in central nervous system regulation via the highly interconnected gut-brain axis affecting microglia indirectly by influencing the structure of the gut-brain axis or directly affecting microglia function and activity. Accordingly, pathological changes in the central nervous system are connected with changes in intestinal metabolite levels as well as altered microglia function and activity, which may contribute to the pathological process of each neuroinflammatory condition. Here, we discuss the mechanisms by which gut metabolites, for instance, the bile acids, short-chain fatty acids, and tryptophan metabolites, regulate the structure of each component of the gut-brain axis, and explore the important roles of gut metabolites in the central nervous system from the perspective of microglia. At the same time, we highlight the roles of gut metabolites affecting microglia in the pathogenesis of neurodegenerative diseases and neurodevelopmental disorders. Understanding the relationship between microglia, gut microbiota, neuroinflammation, and neurodevelopmental disorders will help us identify new strategies for treating neuropsychiatric disorders.

## 1. Introduction

There is a complex and diverse symbiotic microbial system in the human gut [1], which results in innumerable biochemical reactions and the production of diverse intestinal metabolites including bile acids, short-chain fatty acids, branched-chain amino acids, trimethylamine N- oxide, and tryptophan, indole derivatives among many others. Well-characterized bidirectional communication channels exist between the gut and brain, involving neural, endocrine, and inflammatory mechanisms [2], which collectively constitute the gut-brain axis. The communication between the enteric and central nervous systems, as an important link between the gut and the brain, is mediated and regulated by the vagus nerve. The dynamic gut and blood-brain barriers act as major guards against infiltration. In the healthy state, both barriers are tight and prevent microbiome-related immune signaling to the brain [3].

Previous studies have demonstrated that these important structures in the gut-brain axis such as the blood barriers and vagus nerve act on the brain’s immune system and microglia. Gut metabolites, produced by the reaction of corresponding intestinal enzymes and intestinal microorganisms, act as an important part of intestinal components that regulate brain microglia. N6-carboxymethyl lysine, a major gut metabolite that can enter the circulation due to impairment of the intestinal barrier, causes an oxidative surge in microglia [4]. By affecting the pathways of microglia, gut metabolites can shape the nervous system and have been proposed as a potential target for the modulation of neurodegenerative diseases. Results from preclinical and clinical studies have opened the possibility of treating microglia-associated neuroinflammation through the gut-brain axis, which is relevant for disorders such as depression, Alzheimer’s disease, and Parkinson’s disease [5, 6]. The roles of the gut flora on microglia have been demonstrated by changing the integrity and permeability of these structures. The changes in gut microbiota composition caused by aging play a key role in coordinating the morphology and function of microglia through the gut-brain axis, changing the body's anti-inflammatory mechanism and making it easier for inflammatory factors to stimulate microglia from a resting state to an active state [7].

This article reviews the indirect effects of various intestinal metabolites on microglia through the regulation and alteration of major structures constituting the gut-brain axis as well as their direct effects on microglia. On this basis, we take intestinal metabolites as the entry point to summarize the mechanism of intestinal metabolites acting on the corresponding structures of the gut-brain axis as well as their effects on the activity and functional status of microglia, we then highlight the role of the connection between microglia and gut metabolites in neuroinflammation and neurodevelopmental disorders. This knowledge can enrich our understanding of the link between gut metabolites and microglia, facilitating the discovery of novel therapeutic targets for neurological and psychiatric disorders.

## 2. Gut metabolites indirectly affect microglia via the structure of the gut-brain axis

Gut metabolites are substances produced by biochemical reactions in the intestine, among which short-chain fatty acids (SCFAs), bile acids, tryptophan metabolites, branched-chain amino acids, trimethylamine N-oxides, indole derivatives, etc., which also play a crucial role in the body. Tyrosine derivatives interfere with oligodendrocyte maturation and myelination, thus affecting neural development [8, 9]. Conversely trimethylamine-N-oxide, 5-amino valeric acid, 5-aminolevulinic acid betaine, imidazole nic acid, and hippuric acid can promote axonogenesis both in vitro and in vivo [10]. Currently, machine learning algorithms can predict individual metabolite levels based on host genetics, gut microbiome, clinical parameters, diet, lifestyle, and other parameters, which makes it possible to adjust metabolite levels and study the effects of different metabolite groups on the body [11].

Microglia, along with perivascular macrophages (pvMFs) and meningeal macrophages (mMFs) in the central nervous system (CNS), are currently thought to be the only macrophages that survive in the adult brain as remnants of early primitive hematopoiesis [12-14]. Microglia are the major brain-resident macrophages that act as the first line of defense against injury and infection in the CNS and are involved in countless processes, including brain function, brain development, and immune responses [15]. Throughout their life cycle, self-renewing and long-living microglia are able to rapidly adopt context-dependent activation states, which are defined by different transcriptomic features. Thus, microglia also have different physiological functions during development and adulthood, ranging from an active state to a homeostatic state [16]. Due to their close contact with other cells in the CNS, microglia are also involved in the pathogenesis and modulation of many neuroinflammatory and neurodegenerative diseases [17, 18]. During aging, microglia switch from a resting state to an activated state which induces microglial branching, monitoring, and interleukin-1b release via the two-pore domain K+ channel THIK-1, contributing to the development of neurodegenerative diseases [19, 20]. In recent years, the concept of the gut-brain axis has increasingly linked the gut to the nervous system and immune system in the brain. As components of the gut-brain axis, gut metabolites are linked to the CNS and have been discussed in the context of the regulation of astrocyte activity in previous studies. We summarized their relationship from the perspective of microglia [21]. The indirect effects and influence of intestinal metabolites on microglia will be discussed in this section (Table 1).

### 2.1. Gut metabolites affect the permeability and integrity of the gut-blood barrier

The multilayered gut-blood barrier (GBB), which includes the mucous layer, epithelial layer, basement membrane, and vascular endothelium [22], serves as a protective barrier that prevents pathogenic microorganisms, luminal proinflammatory factors, and luminal antigens from entering the bloodstream [23].

The GBB acts as a significant link by controlling metabolites in the gut that pass through the blood to the brain and serves as a dividing line along the gut-brain axis, separating direct communication between the brain and the gut. Therefore, changes in the integrity and permeability of the intestinal blood barrier may also have certain effects on the brain.

Bruce et al. analyzed fecal microbiota from 50 patients who reported no symptoms of gastrointestinal physiological disorders, 22 of whom met the criteria for a psychiatric diagnosis of depression or anxiety disorder (DEP/ANX) while the remaining 28 were healthy controls. Compared with the microbiome of healthy controls, the data analysis uncovered significant gut dysbiosis in the DEP/ANX patients. Of particular concern was the plethora of LPS biosynthetic genes in the gut microbiome of DEP/ANX patients. The overexpression of genes from these KEGG categories in DEP/ANX microbiota also reflected the harmful downregulation of the protective glycosaminoglycan mucin and the emotional neurotransmitter pathways, but not the beneficial counterparts in controls. This suggests that the integrity and permeability of the intestinal barrier may be related to anxiety and depression disorders [24].

**Table 1:** Gut metabolites act on the gut-brain axis structure to indirectly regulate microglia.

Intestinal metabolites	Structure	Mechanism	Reference	
**N6-carboxymethyl lysine**	Gut-blood barrier	changes the integrity of the intestinal barrier and increases the oxidation of microglia	(4)
**acetic acid, propionic acid**	Gut-blood barrier	increases the transepithelial resistance of Caco-2 intestinal epithelial cells and stimulates the formation of tight junctions by inhibiting NLRP3 inflammasome and autophagy	(38)
**butyrate**	Gut-blood barrier	affects oxygen consumption in epithelial cells and leads to the stabilization of hypoxia-inducible factor	(39)
**butyric acid**	Gut-blood barrier	increase the expression of antimicrobial peptide LL-37 in the colon	(40-42)
**bile acids**	Gut-blood barrier	activates the TGR5 receptor located on endocrine L cells of the ileum and colon, induces secretion of glucagon-like peptide, and promotes the regeneration and repair of intestinal epithelial cells	(46-48)
**lipopolysaccharide**	Gut-blood barrier	1. induces the production of NF-κB and pro-inflammatory IL-8 in HEK-TLR4 cells, while TAK-1 can activate IKK and MLCK/MYLK genes to change intestinal epithelial permeability 2. Induced intestinal injury is associated with disruption of antioxidant homeostasis, leading to mitochondrial dysfunction and mitophagy	(49-51)
**dietary tryptophan**	Gut-blood barrier	Inhibits the NF-κB-Myosin light chain kinase signaling pathway, activates the extracellular regulated protein kinase (ERK) 1/2 mitogen-activated protein pathway, and restores intestinal barrier integrity	(51)
**anthocyanin-3-glucosides**	Gut-blood barrier	through its antioxidant capacity, inhibit epithelial inflammation and affects cell apoptosis	(52)
**indole metabolites**	Gut-blood barrier	activates the aromatics receptor, IL-10R1 is significantly induced in cultured intestinal epithelial cells, thereby regulating mucosal integrity	(53)
**vitamin B12**	Gut-blood barrier	plays a protective role during intestinal injury	(54)
**acetate, propionic acid**	Blood-brain barrier	has a potential effect on increasing the permeability of BBB	(69)
**butyrate**	Blood-brain barrier	decreases the expression of BBB-related tight junction proteins such as ZO-1, Occludin, and Claudin-4 in PSCI mice	(70)
**p-cresol glucuronide**	Blood-brain barrier	plays an indirect role in regulating the whole brain transcriptome and the integrity of the BBB in vivo	(72)
**trimethylamine N-oxide**	Blood-brain barrier	enhances the integrity of the BBB and protects it from inflammatory damage by tightly linking the regulatory protein annexin A1	(73)
**trimethylamine**	Blood-brain barrier	impairs BBB function and disrupts tight junction integrity	(73)
**cholic acid**	Blood-brain barrier	increases the transepithelial resistance and γ-glutamyltransferase activity, whereby BBB function of the neurovascular unit is significantly improved	(74)
**Tauroursodeoxycholic acid**	Blood-brain barrier	decreases BBB permeability and improves nerve function and cerebral blood flow in SAH mice	(75)
**Deoxycholic acid, ursodeoxycholic acid**	Blood-brain barrier	affects the integrity of the BBB	(76-77)
**caffeic acid**	Blood-brain barrier	1.significantly reduces the serum levels of S100β, a marker of brain damage 2.upregulates ZO-1 tight-linking protein levels during stroke in obese mice	(78)
**butyrate**	Vagus nerve	when the dose is increased, it has a hypotensive effect on the VN	(89)
**SCFAs**	Vagus nerve	can signal through multiple GPCRs including hydroxycarboxylic acid receptor 2, olfactory receptor 78, as well as fatty acid receptors 2, and fatty acid receptors 3	(91)
**5-Hydroxytryptamine**	Vagus nerve	activates 5-HT3 receptors on afferent fibers of the VN	(92)
**tryptophan catabolites**	Vagus nerve	activates the Trpa1 channel on intestinal endocrine cells, causing rapid activation of intestinal and vagus nerve cells by secreting the neurotransmitter 5-HT	(93)
**indole**	Vagus nerve	alters colonic vagal afferent activity by modifying GLP-1 regulation	(94)
**γ-aminobutyric acid**	Vagus nerve	plays a crucial role in the signaling function of the VN	(95)
**Hydrogen sulfide**	Vagus nerve	stimulated by targeting the TRPV1 receptor in intestinal afferents	(96)

In the context of MS, "classically activated" microglia are thought to be critical for myelin phagocytosis, antigen presentation to T cells, and release of pro-inflammatory cytokines release in active lesions [25]. In addition, reactive microglia have long been considered a contributing factor in Parkinson’s disease [26]. In Parkinson’s disease and multiple sclerosis, common diseases associated with microglia, a correlation has also been demonstrated with intestinal barrier integrity and permeability [5, 27]. Moreover, research has suggested that age-induced alterations in the microbiota, can disrupt the integrity of the gut barrier, and promote N6-carboxymethyl lysine accumulation in the brains of aged mice and humans, leading to an oxidative surge in microglia. Accordingly, it was found that intestinal barrier damage may make it easier for some substances that can cross the blood-brain barrier to cross the intestinal barrier and reach the brain [4]. In an experimental acute colitis model induced by dextran sodium sulfate in drinking water, researchers observed intestinal myeloid cell infiltration, impaired intestinal barrier, elevated frequency of inflammatory M1-like microglia, as well as augmented release of proinflammatory cytokines. In contrast to acute colitis, there is a lower degree of intestinal myeloid cell infiltration and fewer intestinal barrier changes, as well as an increased proportion of repaired M2-microglia in the hippocampus, and higher levels of anti-inflammatory cytokines IL-10 and IL-10 [28]. Therefore, it can be inferred that the permeability and integrity of the GBB may have a potential relationship with the state of microglia, which may have a certain impact on the normal nervous system function. Protein tyrosine phosphatase 1B (PTP1B) is an important mediator of crosslinking neuroinflammation and synapse impairment [29, 30]. lipopolysaccharide can promote the inflammatory neurodegenerative cascade in the brain of fiber-deficient mice via the PTP1B-PcamKII-PgSK3β pathway, which is related to the impaired gut blood barrier, resulting in abnormal tau phosphorylation and harmful activation of microglia [31, 32]. After the integrity and permeability of the GBB are disrupted, substances in the gut may affect microglia through this pathway. Other specific mechanisms have not yet been proved by sufficient experimental evidence, but they merit further study in the future.

**Figure 1.**
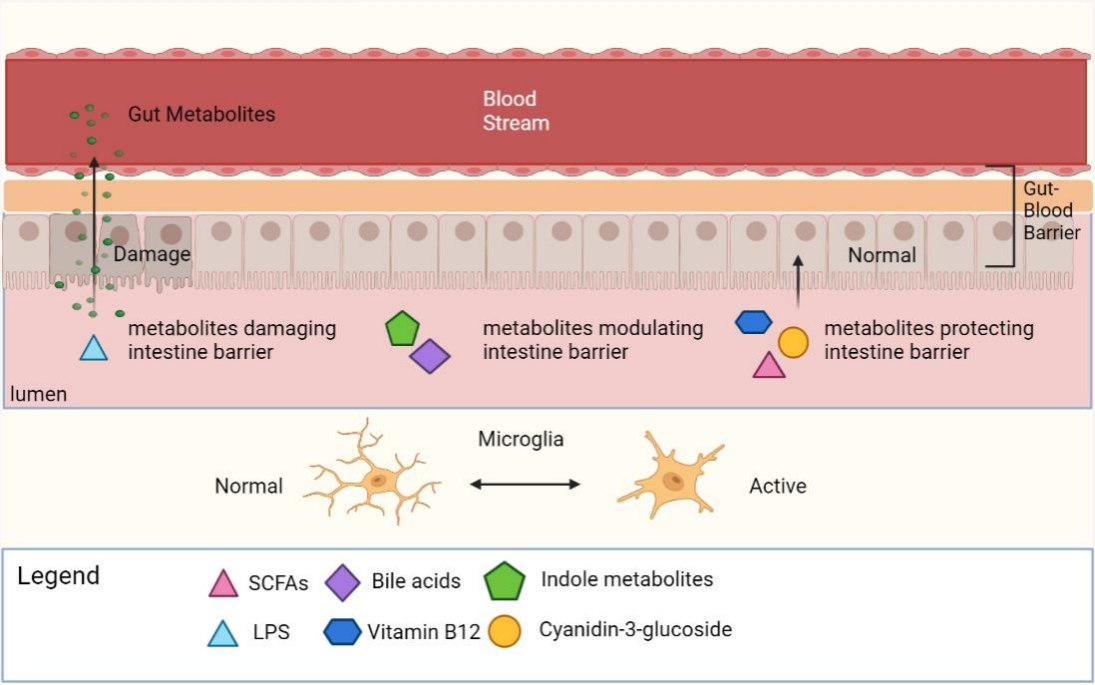
**Gut metabolites regulate the permeability and integrity of the GBB**. GBB is thought to be a significant link in the gut-brain axis, which controls metabolites from the gut via the blood to the brain. Some intestinal metabolites regulate the status of microglia indirectly by changing the integrity and permeability of the GBB, thereby affecting the communication between the gut and the brain. Abbreviation: SCFAs, short-chain fatty acid. LPS, lipopolysaccharide. (Figure created with Biorender.com).

The functional status of the major pathways of endothelial and epithelial barrier flow is partially regulated, at the level of intercellular tight junctions, which are complex and dynamic structures [33], which can be influenced by various factors. Environmental stimuli can lead to an imbalance of the microbiome, triggering zonulin release and increasing the inflow of antigens from the intestinal lumen into the lamina propria, which activates the immune system, leading to the release of IFN-γ and TNF-α, further exacerbating the increase of intestinal permeability and immune activation [34].In addition to the dysbiosis of the gut flora, intestinal metabolites are also key factors affecting the integrity and permeability of the GBB (Fig. 1).

Depending on the amount of protein digested, 6-18 g of nitrogenous material enters the lumen of the large intestine daily via the ileocecal junction. These materials are used as a substrate by the microbiota, which finally forms a complex mixture of metabolites, including short-and branched-chain fatty acids, hydrogen sulfide, ammonia, aminophenol, indole, and N-nitroso compounds. The beneficial and detrimental effects of these compounds on the colonic epithelium depend on parameters such as their luminal concentration, the duration of colonic stasis, and other factors [35].

SCFAs are crucial gut metabolites and many studies have suggested that they have a protective role on the intestinal epithelial barrier. Fernando et al. used an antibiotic-induced bacterial depletion mouse model to determine the role of gut microbiota and their molecular mediators in the gastrointestinal tract, which revealed that SCFAs restored the antibiotic-induced increase of intestinal permeability [36]. The study demonstrated that the microbial SCFAs protect intestinal barrier integrity in mouse intestinal epithelial cells (IEC) by promoting IEC RegIIγ and β-defenses in a GPR43-dependent manner via the activation of the mTOR pathway and STAT3 phosphorylation. Similar results were obtained in human colonic epithelial cell lines [37]. In addition, SCFAs produced by Lactobacillus strains such as acetic acid, propionic acid, and butyrate can increase the transepithelial resistance of Caco-2 intestinal epithelial cells and stimulate the formation of tight junctions by inhibiting the NLRP3 inflammasome and autophagy[38]. Kelly et al. sought that bacterial-derived butyrate affects oxygen consumption by epithelial cells and leads to the stabilization of hypoxia-inducible factor, which promotes epithelial barrier function [39]. Regulating the thickness of the mucous layer that protects the mucosa is also an important mechanism involved in epithelial barrier function. SCFAs modulate the secretion of antimicrobial peptides by the gut epithelium to enhance the epithelial barrier function. Among them, butyric acid can increase the expression of antimicrobial peptide LL-37 in the colon [40-42]. By contrast, 27-hydroxycholesterol was found to induce inflammatory changes in the gut and circulation as well as alterations in the gut microbiome and SCFAs, disrupting the intestinal barrier [43]. However, recent studies were unable to clearly elucidate the appropriate dosage and concentration of SCFAs for optimal intestinal barrier function. At the same time, the specific effects of each SCFA have not been conclusively studied although the link between SCFAs and the intestinal barrier was widely demonstrated. Moreover, the lack of relevant clinical trials also hinders the development of treatments based on SCFAs. However, changing the homeostasis of SCFAs to improve the GBB function can indirectly regulate the phenotype and activity of microglia, which may also be a viable strategy for the treatment of neurological and psychotic disorders in the future.

Bile acids (BAs) are emulsifiers that can break down large globules of dietary fat into tiny droplets, but high concentrations of BAs can destroy membranes due to these same detergent properties [44, 45]. TGR5 receptors located on gut endocrine L cells in the ileum and colon are activated by different concentrations of BAs to secrete glucagon-like peptides, which promote the regeneration and repair of intestinal epithelial cells [46-48].

Lipopolysaccharide (LPS), a highly immune-stimulatory component of the outer membrane in Gram-negative bacteria, also affects the gut barrier. Studies have shown studies that HEK-TLR4 cells could be induced by LPS to produce NF-κB and proinflammatory IL-8 whereby the IKK and MLCK/MYLK genes were activated by TAK-1 to alter intestinal epithelial permeability [49, 50]. In addition, LPS-induced intestinal injury is associated with disruption of antioxidant homeostasis, which results in mitochondrial dysfunction and triggers mitochondrial dysfunction. In the face of LPS-induced TJ injury, dietary tryptophan can inhibit the NF-κB-Myosin light chain kinase signaling pathway, activate the extracellular regulated protein kinase (ERK) 1/2 -mitogen-activated protein pathway, and restore intestinal barrier integrity [51].

Anthocyanin-3-glucosides and their phenolic metabolites produced in the gut protect the intestinal barrier from intestinal damage by their antioxidant effects, inhibition of epithelial inflammation, and influence on apoptosis [52]. Administration of indole metabolites resulted in prominent induction of IL-10R1 on cultured intestinal epithelial cells due to aryl hydrocarbon receptor activation, thereby modulating mucosal integrity [53]. The study pointed out that vitamin B12 has a certain protective effect on the intestinal epithelium in the state of intestinal damage [54]. The repair and protection of the intestinal barrier by these dietary intestinal metabolites may be a therapeutic approach for protecting the gut-brain axis and indirectly regulating microglia in the future.

### 2.2. Gut metabolites affect the permeability and integrity of the blood-brain barrier

Blood vessels mainly consist of endothelial cells (ECs) that make up the walls of the blood vessels, and mural cells that lie on the abluminal surface of the EC layer [55]. The presence of tight junctions between endothelial cells, the near absence of pinocytosis, and the absence of fenestra prevent ultrafiltration in the capillary bed of the brain, thus realizing the barrier function of the blood-brain barrier (BBB) [55-57]. Functional changes of tight junctions caused by inflammation induce a breakdown of the BBB, which promotes paracellular leakage, inducing cysts, thereby promoting extracellular leakage. The properties of the BBB are primarily regulated by ECs but are induced and maintained through critical interactions with parietal cells, immune cells, glial cells, and nerve cells in the neurovascular unit [57].

Microglia have a certain role in regulating the integrity and permeability of the BBB. Forsberg et al. found that IBA-1-positive microglia co-localized with the polysaccharide calyx marker Ulex Europaeus lectin 1 (UAE-1) in fine blood vessels, suggesting that activated microglia could interact with the vascular polysaccharide calyx to disrupt vascular integrity [58], In addition, rodent models of cardiac arrest and subsequent cardiopulmonary resuscitation demonstrated that disruption of the endothelial glycocalyx leads to increased BBB permeability [59]. Using extravascular fibrinogen as a marker of vascular disruption, researchers found that chronic mild hypoxia triggered transient vascular leakage in spinal cord blood vessels, particularly in the white matter, which was associated with clustering and activation of Mac-1-positive microglia around disrupted vessels [60]. Further mechanistic studies revealed that microglia-mediated activation of matrix metallo-proteinases-2 and metalloproteinases-9 contributed to BBB disruption in a rotenone-induced mouse model of Parkinson’s Disease [61]. A recent study demonstrated that microglial cells activated by LPS may promote invasion and barrier dysfunction in brain microvascular endothelial cells by modulating the CXCL13/CXCR5 axis and p38 signaling [62]. In addition to damaging the BBB under pathological conditions, microglia can also protect it. A study investigating four types of multiple sclerosis plaques found that the microglial miR-126a-5p is positively correlated with BBB integrity. Mechanistically, the loss of miR-126a-5p in microglia exacerbates BBB leakage [63]. Il-10 receptor-mediated microglial function reduces inflammation and induces damage repair. Increased IL-10 levels in the brain activate IL-10R1 and IL-10R2, triggering the JAK1 pathway and promoting the translocation of STAT3 to the nucleus of microglia. This mechanism inhibits the secretion of proinflammatory cytokines and ensures the polarization of microglia toward the M2 phenotype [64, 65].

**Figure 2.**
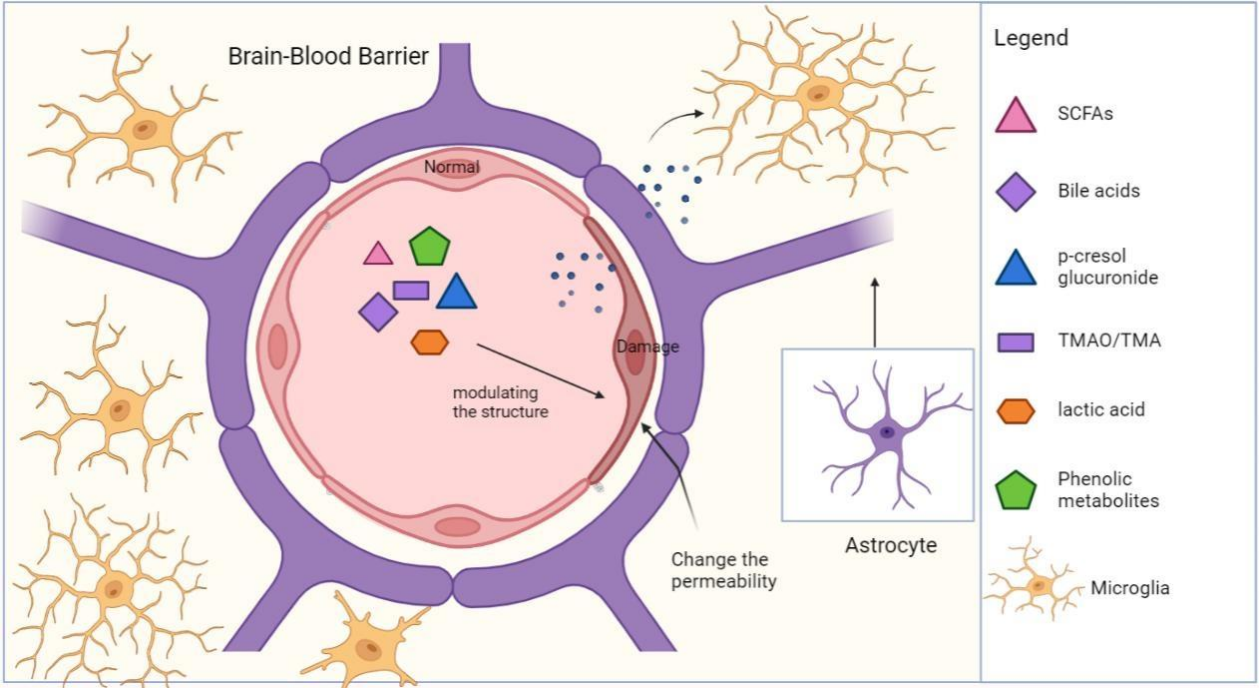
**Gut metabolites regulate the permeability and integrity of the BBB**. The BBB inhibits the passage of most molecules produced by microbes from circulation into the CNS. Metabolites can alter the integrity of the BBB, thereby increasing translocation and permeability, making the barrier function of the BBB decrease. At the same time, changing the integrity and permeability of the blood-brain barrier will affect the function of microglia. (Figure created with Biorender.com).

As a protective defense line of the CNS, the integrity and permeability of the BBB enable it to maintain brain homeostasis. At the same time, as a significant link in the gut-brain axis, it can restrict the entry of intestinal metabolites and inflammatory factors into the CNS, thus preventing the activation of microglia, and resulting neuroinflammation. The permeability and integrity of the BBB affect the activity of microglia. The increased inflammatory response in microglia was found to be closely correlated with increased peripheral inflammation, a loss of BBB integrity, and infiltration of immune cells in the brain parenchyma in a mouse model of telomere shortening [66]. Interleukin-1 signaling in BBB-ECs upregulate the expression of the adhesion molecules Vcam-1, Icam-1, and the chemokine receptor DARC, all of which have been previously shown to promote CNS-specific inflammation [6]. Intestinal metabolites have a significant influence on the permeability of the BBB. Following 24 h after a single LPS injection, the highest dose of LPS (3mg/kg) administered to CD-1 mice significantly increased BBB permeability (as measured using 14 C-sucrose, with lower levels indicating increased peripheral vascular bed permeability), whereas the lower two doses had no effect. LPS increased Iba1 and F4/80 staining, while the Iba1 IHC results showed that indomethacin did not block this effect, suggesting increased microglia/macrophage activation [67]. Gut metabolites are one of the main factors regulating the integrity and permeability of the BBB (Fig. 2).

SCFAs were shown to be probable regulators of BBB integrity in GF mice [68]. In rhesus monkeys, the use of antibiotics to induce changes in gut microbial composition, particularly a reduction in acetate and propionic acid-producing phyla and genera, was found to potentially increase BBB permeability [69]. Researchers transferred gut microbiota from post-stroke cognitive impairment (PSCI) patients into stroke model mice. Compared with mice receiving FMT from non-PSCI patients, the stroke model mice receiving FMT from PSCI patients had a higher abundance of Enterobacteriaceae, lower fecal butyrate levels, and significantly reduced expression of tight junction proteins associated with the BBB, such as ZO-1, Occludin, and Claudin-4 [70]. Using immunofluorescence staining, the authors observed prominent microglial activation in the hippocampi of PSCI mice [70]. These studies illustrated the effects of SCFAs on the BBB through both direct and indirect, corroborating the effects of gut metabolites on the BBB.

Inhibition of lactate production in the endothelium by deletion of the glucose transporter-1 (GLUT1) in mice leads to a breakdown of BBB and increased permeability due to the loss of pericyte coverage in the retina and brain vasculatures [71]. Systemically circulating p-cresol glucuronide (PCG) is a gut metabolite that has an indirect effect on the regulation of the BBB. In vivo, PCG was found to regulate the integrity of the BBB and affect the whole brain transcriptome, while in vitro, PCG had little effect on endothelial cells and did not affect the expression of the key tight junction molecule occluding [72]. Recent studies, using an integrated in vitro/in vivo approach, have shown that the choline metabolite trimethylamine n-oxide (TMAO) when provided in the diet at physiologically relevant concentrations enhances the integrity of the BBB as well as protecting it from inflammatory damage via the tight junction regulatory protein annexin A1. By contrast, trimethylamine (TMA), a precursor of TMAO, impairs BBB function and disrupts tight junction integrity [73].

In addition to choline metabolites, BAs also regulate the integrity of the BBB. One study showed that cholic acid (CA) treatment increased transepithelial electrical resistance (TEER) and γ-glutamyltransferase activity while decreasing the permeability coefficient of sodium fluorescein compared with the untreated model group. These results indicated that CA treatment achieved a significant improvement in neurovascular unit (NVU) BBB function after oxygen-glucose deprivation reoxygenation (OGD/R). In addition, CA has also been shown to have a significant protective effect on neurons [74]. The extravasation of Evans blue was significantly reduced in mice pretreated with tauroursodeoxycholic acid (TUDCA) at 24 hours after SAH compared with the control group. TUDCA significantly reduced BBB permeability while improving neurological function and cerebral blood flow in SAH mice [75]. In addition, deoxycholic acid and ursodeoxycholic acid also affect the integrity of the BBB [76, 77]. In addition to bile acid metabolites, some phenolic metabolites also affect the BBB. In mice fed a high-fat diet (HFD) treatment with caffeic acid or Antirhea borbonica polyphenols significantly reduced the serum levels of S100β, a peripheral biomarker of brain injury and BBB permeability. In addition, caffeic acid increased the ZO-1 tight junction protein levels in obese mice during the stroke. These findings suggest that caffeic acid can ameliorate hemorrhagic transformation and protect the BBB in a mouse model of stroke exacerbated by obesity [78]. The effects of intestinal metabolites on the BBB have been verified in a number of disease models, and are often associated with microglia. Drugs targeting these intestinal metabolites may gradually become a research hotspot in the regulation of neuroinflammation and other diseases via the BBB.

### 2.3. Gut metabolites act as signaling molecules that activate the vagus nerve

The brain and the gut are not adjacent organs, and they need certain pathways to transfer neural or biochemical signals. Intestinal flora can potentially regulate the gut-brain axis through a variety of direct and indirect pathways. They include immune (cytokine), neural (vagus and enteric nervous system), and endocrine (via the hypothalamic-pituitary-adrenal axis) pathways [79]. The enteric nervous system, consisting of two layers of ganglia and fibers surrounding the gastrointestinal tract, provides the intrinsic innervation of the bowels and is the most diverse neurochemical component of the peripheral nervous system [80]. The vagus nerve (VN), a significant part of the gut-brain axis, connects the enteric nervous system with the CNS. It is composed of 80% afferent and 20% efferent fibers [81], which innervate structures in the head, neck, thorax, and abdomen [82]. While belonging to the parasympathetic division of the autonomic nervous system, the function of this nerve is primarily sensory, and it is dominated by sensory axons [82].

The VN has been demonstrated to modulate both microglial activation and peripheral immune responses [83-85]. Lactobacillus rhamnosus JB-1 induced a decrease in hippocampal IBA-1-positive microglia, but vagotomy converted this into an increase in hippocampal IBA-1-positive microglia, which supports the relationship between the VN pathway and microglia [86]. The gut microbiota of sepsis patients can be altered by fecal microbiota transplantation (FMT). FMT-treated LPS mice had better spatial memory ability, less abnormal electroencephalography findings, significantly reduced levels of some inflammatory factors, and decreased amounts of IBA-1-positive microglia in the cortex. This suggests that the VN is a key mediator of the relationship between gut microbiota and sepsis-associated encephalopathy [87]. In rats with transient middle cerebral artery occlusion, berberine mediated microglial regulation by vagus stimulation via hydrogen sulfide, a metabolite derived from gut microbes [88]. Neurons constitute a crucial pathway of the gut-brain axis, and intestinal metabolites can regulate the function of microglia through this indirect effect. The VN may change the status and quantity of some factors in the brain, such as pro-inflammatory cytokines or some stimulus priming factors, through the reduction of certain signal inputs, leading to a change in the microglial state. However, the specific mechanism and effect still need to be elucidated in future studies.

**Figure 3.**
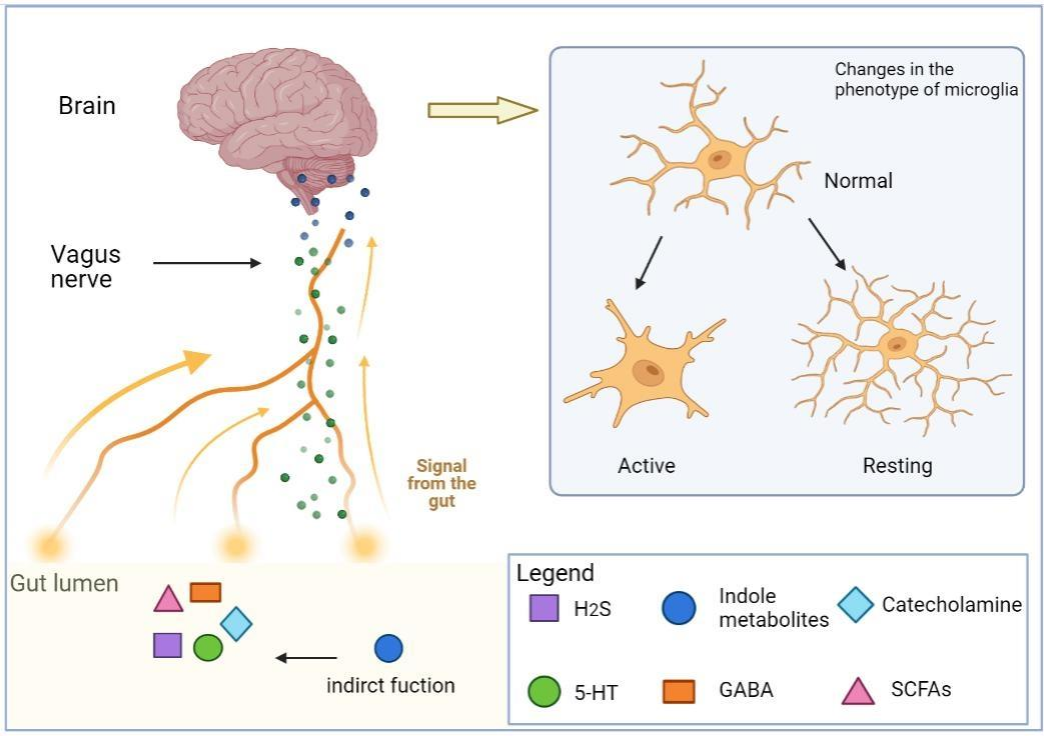
**Gut metabolites regulate vagus function and state**. The VN is a crucial part of the gut-brain axis, connecting the enteric nervous system with the CNS. Communication between the gut and the brain is closely connected with the function and signaling of the VN. In the gut, many bacterial metabolites can act as signaling molecules to directly regulate vagal nerve activity or indirectly affect vagal nerve function. Ultimately, the phenotype of microglia is altered Abbreviation: SCFAs, short-chain fatty acid. 5-HT, 5-hydroxytryptamine. GABA, γ-aminobutyric acid. H2S, Hydrogen sulfide. (Figure created with Biorender.com).

Some intestinal metabolites can activate the VN via the enteric nervous system (Fig. 3). One study found a significant hypotensive effect of butyrate when the administered dose increased the local concentration in the colon by a factor of 2 to 3. Hypotensive and bradycardic responses were significantly reduced by subphrenic vagotomy, suggesting that the hypotensive regulatory effect of butyrate may be mediated by the VN [89]. Gut-derived molecules can directly modulate the activity of vagal afferents through g-protein receptors (GPCRs) [90]. The SCFAs acetate, propionate, butyrate, and valerate can signal through multiple GPCRs including hydroxycarboxylic acid receptor 2, olfactory receptor 78, as well as free fatty acid receptors 2, and fatty acid receptors 3 [91]. This once again demonstrates that SCFAs act as signaling molecules for the VN. In addition to SCFAS, some amino acid metabolites have the same effect on the VN. For example, 5-hydroxytryptamine (5-HT), synthesized from tryptophan and released in response to various stimuli, is abundant in enterochromaffin cells. It has been demonstrated that 5-HT released from enterochromaffin cells activates 5-HT3 receptors on afferent fibers of the VN [92]. Tryptophan catabolites produced by gut bacteria, such as indole and indole acetaldehyde activate Trpa1 channels on enteroendocrine cells (EECs), causing rapid activation of enteric and vagal neurons through secretion of the neurotransmitter serotonin (5-HT) [93]. Glucagon-like peptide-1 (GLP-1), a neuroregulatory molecule, is secreted by intestinal endocrine L-cells in the colon. The bacterial metabolite indole can alter colonic vagal afferent activity by modifying GLP-1 regulation [94]. In addition, γ-aminobutyric acid A receptors also play a crucial role in the signaling function of the VN [95]. Hydrogen sulfide (H2S), a gaseous transmitter, is produced by gut bacteria and epithelial cells. The VN is stimulated by targeting transient receptor potential vanillic acid 1 (TRPV1) in intestinal afferents [96]. Electrical VN stimulation attenuates tumor necrosis factor (TNF) production during sepsis through the cholinergic anti-inflammatory pathway, which depends on the integrity of the VN and catecholamine production [97]. It stands to reason that these gut metabolites could act as neurotransmitters to affect the VN or regulate the concentration and quantity of related neurotransmitters, ultimately affecting the function of microglia.

## 3. Gut metabolites directly affect the function and activity of microglia

Microglia are an important part of the gut-brain axis, many studies have demonstrated that gut metabolites are able to directly affect the function and activity of microglia (Table 2).

SCFAs, which are among the many metabolites released by gut microbes, can cross the BBB alone or in a mixture to interact with microglia and regulate their homeostasis, Accordingly mice lacking the SCFA receptor FFAR2 mirrored microglial defects found under GF conditions [98, 99]. Sodium butyrate exacerbates the inflammatory response by upregulating the expression of inflammatory mediators in BV2 microglial cells stimulated by LPS [100]. In GF animals, acetate affects the metabolic profile of microglia and promotes their maturation, inhibiting cellular phagocytosis in the steady state and ultimately leading to an increased burden of Aβ in 5xFAD animals [101]. At the same time, acetate induced a proinflammatory phenotype of microglia and increased cytokine expression, which in turn attenuated phagocytosis [102]. In addition to accelerating the occurrence and development of some diseases by regulating the function of microglia, SCFAs can also prevent or treat some neurological diseases by inhibiting the activity of microglia [103]. Fragas et al. demonstrated that acetate could play an antagonistic role in the cellular inflammatory profile depending on the time of administration [104]. Clostridium butyricum (CB) treatment has been shown to prevent cognitive dysfunction, Aβ deposition, microglia activation, as well as the production of interleukin (IL) -1β and tumor necrosis factor (TNF) -α in the brains of APP/PS1 mice. At the same time, the dysregulation of intestinal microflora and butyrate was reversed after CB treatment. Notably, butyrate treatment reduced the Aβ-induced levels of CD11b and COX-2, while inhibiting the phosphorylation of NF-κB p65 in BV2 microglia [105]. In addition to short-chain fatty acids, the long-chain fatty acids 10-oxo-trans-11-octadecenoic acid and 10-hydroxy-cis-12-octadecenoic acid generated from linoleic acid by the gut lactic acid bacterium Lactobacillus plantarum also inhibit the activation of microglia [106]. Recent studies have gradually revealed that different concentrations of SCFAs have different effects on microglia, while some long-chain fatty acids also have a certain effect. However, the specific mechanism of action is still uncertain. For example, the finding of increased histone acetylation by acetic acid in T cells, since their mediating influence cannot be excluded, leads to the conclusion that acetic acid is involved in microglia metabolism, but this hypothesis requires further studies [107]. Moreover, there are few studies on the metabolic changes of microglia in vivo and most are based on in vitro experiments. In the future, it will be necessary to explore their specific regulatory effects on microglia.

**Table 2:** Gut metabolites that act directly on microglia.

Intestinal metabolites	Structure	Mechanism	Reference
**sodium butyrate**	Microglia	upregulates the expression of inflammatory mediators in BV2 microglia and aggravates the inflammatory response	(100)
**acetate**	Microglia	1. affect the metabolic characteristics of microglia and promotes their maturation, while also inhibiting phagocytosis in the steady state 2. Induces the proinflammatory phenotype of microglia and increases cytokine expression 3.plays an antagonistic role in the cellular inflammatory profile depending on the time of administration	(101-104)
**butyrate**	Microglia	reduce the Aβ-induced levels of CD11b and COX-2 , while also inhibiting the phosphorylation of NF-κB p65 in BV2 microglia	(105)
**long-chain fatty acids**	Microglia	inhibits the activation of microglia	(106)
**lipopolysaccharide**	Microglia	1. increases NF-κB p65 levels in microglia, promoting gene expression of pro-inflammatory cytokines, making microglia more prone to transition to an inflammatory phenotype 2. promotes the inflammatory and neurodegenerative cascade in the brain	(32) (109)
**ursodeoxycholic acid**	Microglia	reduces the production of proinflammatory cytokines NO and IL-113 in Aβ42-pretreated mouse microglia	(113)
**tauroursodesoxycholic acid**	Microglia	1. mediated by the receptor GPBAR1/TGR5, increases cAMP levels, thereby mediating its anti-inflammatory effects on microglia 2. acts as a glycolytic regulator and attenuates the proinflammatory response of microglia	(114) (119)
**taurocholic acid**	Microglia	acts as a glycolytic regulator and attenuates the proinflammatory response of microglia	(119)
**polyphenols**	Microglia	acts against LPS-induced inflammation in BV-2 microglia	(120)
**lignin metabolites**	Microglia	have protective effects in injury induced by the production of NO and pro-inflammatory cytokines (IL-6 and TNF-α) in BV-2 microglia	(120)
**Urolithins**	Microglia	1. reduces the intermediate levels of NO, IL-6, PGE2, and tumor necrosis factor-α in LPS-BV-2 microglia 2. suppresses the gene expression of proinflammatory cytokines and regulated the JNK/c-Jun signaling pathway, promoting M2 microglial polarization	(122)
**indole-3-propionic acid**	Microglia	positively correlated with the serum levels of brain-derived neurotrophic factor (BDNF), reducing the concentration of pro-inflammatory TNF-α in activated microglia	(21) (127)
**NAMO**	Microglia	restores NAD+ -dependent mitophagy and inhibits microglial activation	(128)
**trimethylamine oxidase**	Microglia	increases microglia-mediated neuroinflammation and the production of ROS in the hippocampus	(129)
**Isoamylamine**	Microglia	enhances S100A8 expression, promoting the apoptosis of microglia	(130)

LPS is a unique molecule found in the outer cell membrane of Gram-negative bacteria. When the bacteria die, LPS can interact with animal cells resulting in toxicity. Previous studies have shown that systemic TNFα and LPS activate microglia in wild-type mice and increase the expression of brain proinflammatory factors [108]. In a related in vitro study, LPS increased NF-κB p65 levels in microglia (BV-2 cells), promoting gene expression of pro-inflammatory cytokines, and making microglia more prone to transition to an inflammatory phenotype [109]. Increased LPS in the gut may cross the GBB and BBB, activate neuroinflammation and thus affect the status of microglia. LPS has been reported to promote the inflammation-neurodegenerative cascade via the PTP1B-pCaMKII-pGSK3β pathway in the brains of FD mice. Enhanced PTP1B signaling in FD mice, coupled with phagocytosis of activated microglia, causes synaptic ultrastructural changes in the hippocampus, and ultimately leads to cognitive decline [32]. PapRIV, a quorum sensing peptide (QSP), produced by Bacillus cereus, can cross the GBB and the BBB. Both in vivo and in vitro, PapRIV had an activating effect on BV-2 microglia cells [110].

Bile acids can easily cross the BBB [111]. Guo et al. demonstrate that bile acids inhibit NLRP3 inflammasome activation via the TGR5-cAMP-PKA axis and reduce the production of proinflammatory factors [112]. It was found that ursodeoxycholic acid reduced the production of the proinflammatory cytokines NO and IL-113 in Aβ42-pretreated mouse microglia [113]. The effects of tauro ursodesoxy cholic acid (TUDCA) on microglia are mediated by the bile salt receptor GPBAR1/TGR5. TUDCA activates this receptor, increasing the intracellular cAMP levels, as well as mediating its anti-inflammatory role in microglia. In addition, TUDCA upregulated microglial anti-inflammatory markers and downregulated proinflammatory markers [114]. Similarly, TUDCA supplementation ameliorated neuroinflammation in experimental autoimmune encephalomyelitis (EAE) through its effects on GPBAR1 [115]. Furthermore, bile acids can also regulate the activity and function of microglia through another indirect pathway. There is a high demand for biosynthetic precursors and energy for proinflammatory macrophages, such as amino acids, nucleotides, and lipids. As a result, the metabolic changes that occur in macrophages are similar to the Warburg effect in tumor cells [116, 117]. These alterations include a high glycolytic rate, increased glucose uptake, and upregulated pentose phosphate pathway, in conjunction with a low rate of oxidative phosphorylation through the TCA cycle [118]. Pyruvate kinase, a key glycolytic enzyme, converts phosphoenolpyruvate to pyruvate and ADP to ATP. PKM2, a homodimer, is an isoform that has lost pyruvate kinase activity and acts as a transcription factor that regulates the expression of target genes related to glycolysis and inflammation [117]. Pretreatment with TUDCA or taurocholic acid decreased the expression of PKM2 and its target genes in cultured microglia. This suggests that bile acids can act as glycolytic regulators and attenuate the proinflammatory response of microglia [119]. The pathways and mechanisms of different bile acids in microglia have been elucidated. Based on the available literature, it can be found that bile acids are a potential target for regulating microglia and countering inflammation via gut metabolites. Although most of the published experiments were done on mice, it is believed that the research on drugs targeting the regulation of bile acids will have a certain value in the regulation of neuroinflammation in humans.

Microbial metabolites of polyphenols (PMMs) can pass through the GBB and BBB. Protective effects of parental polyphenols and their corresponding PMMs against LPS-induced inflammation were observed in BV-2 microglia. Among them, the lignin-derived PMMs, equol, and enterolactone had protective effects against injury induced by NO and pro-inflammatory cytokines (IL-6 and TNF-α) in BV-2 microglial cells [120]. Urolithins, ellagitannin-derived phenolic metabolites of gut microbiota, also regulate microglial status by reducing the intermediate levels of NO, IL-6, PGE2, and tumor necrosis factor-α in LPS-BV-2 microglia [121]. After inflammation induced by LPS and ATP stimulation induced inflammation, urolithin A inhibited the expression of proinflammatory cytokine genes and regulated the JNK/c-Jun signaling pathway. UrolithinA also reduced inducible nitric oxide synthase gene expression and promoted M2 microglial polarization [122].

The kynurenine pathway (KP) is a vital tryptophan metabolic pathway that regulates chronic inflammation and depressive symptoms. In particular, indoleamine 2, 3-dioxygenase 1 (IDO1), the rate-limiting enzyme of the KP, is activated by chronic inflammation and contributes to the production of kynurenine [123]. Kynurenine and its metabolites are endogenous ligands of aromatic hydrocarbon receptors (AHRs), which regulate the differentiation of Treg cells, enabling them to induce immune tolerance and prevent excessive inflammation [124]. In addition, neuroactive metabolites of KP are strongly associated with psychiatric disorders. Quinolinic acid is an excitotoxic N-methyl-D-aspartate (NMDA) receptor agonist, while kynurenic acid is a neuroprotective NMDA receptor antagonist whose metabolic disorders have been associated with depression [125, 126]. The dietary tryptophan metabolites indole-3-propionic acid (IPA) and indole-3-aldehyde act as agonists of the aryl hydrocarbon receptor and are also involved in regulating the activation of microglia [21]. Metabolomic analysis revealed that the metabolites of tryptophan were changed in the elderly who ingested probiotic capsules in clinical trials. The content of IPA was increased, and the increase of IPA levels in the probiotic group was positively correlated with the serum brain-derived neurotrophic factor (BDNF). In addition, in vitro treatment with IPA can significantly reduce the concentration of pro-inflammatory TNF-α in activated microglia, which has the effect of protecting microglia from inflammation, thereby promoting neuronal function [127].

NAMO, a nicotinamide oxidation product of gut microbiota, restores NAD+ -dependent mitophagy and inhibits microglial activation. In addition, the depletion of intestine flora by oral antibiotic treatment triggered the overactivation of microglia leading to herpes simplex virus encephalitis (HSE)-like pathology. In the study, NAMO was found to significantly reduce microglia-mediated pro-inflammatory responses in HSE mice treated with antibiotics as well as untreated mice [128]. Trimethylamine oxidase (TMAO), another metabolite produced by gut microbes, is involved in the pathogenesis of numerous diseases by augmenting oxidative stress and inflammation in peripheral tissues. In a clinical study investigating TMAO treatment, plasma TMAO increased before and 1 week after surgery, which further increased microglia-mediated neuroinflammation and the production of ROS in the hippocampus, leading to the aggravation of cognitive dysfunction in the laparotomy group [129]. The gut metabolite isoamylamine(IAA) interacts with the promoter region of the S100 calcium-binding protein A8(S100A8) gene to promote the unbinding of its complementary hairpin structure, thus enabling p53 to access the S100A8 promoter region and enhance its expression, promoting the apoptosis of microglia [130]. In addition, it was also demonstrated that during aging, Abnormal regulation of the microbiota-macrophage-metabolite axis leads to the induction of IAA, damaging the function of microglia and aggravating brain memory loss, which leads to cognitive impairment in mice [130].

## 4. The role and influence of gut metabolites in neuroinflammation and neurodevelopmental disorders

Since microglia have a wide range of physiological functions in brain homeostasis and development, they are thought to be involved in the pathogenesis of a variety of neuroinflammatory diseases and neurodevelopmental disorders. As we discussed earlier, metabolites produced in the gut have been shown to affect microglia function and activity. Here, we briefly discuss the potential role of gut metabolism-microglia connectivity in neuroinflammatory diseases and neurodevelopmental disorders.

### 4.1. Neuroinflammatory diseases

#### 4.1.1 Alzheimer’s disease (AD)

AD is an unremitting, neurodegenerative disorder characterized by extracellular plaques containing β-amyloid (Aβ) and tau-containing intracellular neurofibrillary tangles [131]. These accumulations result in neuronal cell damage affecting wide areas of the cerebral cortex and hippocampus [132]. Symptoms include language impairments, memory difficulties, psychological and spiritual changes, as well as impairments in activities of daily living [133].

AD risk genes are selectively or preferentially expressed in microglia and show lower expression in other cell types in the brain [134]. β-amyloid (Aβ) binds to lipoproteins, and this complex is efficiently eliminated by microglia in a TREM2-dependent manner [135]. Beyond Aβ clearance, another important function of TRE2-dependent microglia is to remove debris from damaged or dead cells to maintain a healthy brain environment, as observed in models of demyelination or ischemic stroke [136, 137]. Moreover, microglia are the main source of C1q and may play a key role in complement-mediated loss of synapses in AD [138]. Microglia also play an important role in limiting Aβ plaque growth and plaque-associated disruption of neuronal connections [139]. TREM2 protects against AD by enabling microglia to surround and alter Aβ plaque structures, thereby limiting neurite damage [140].

Given the link between microglia and gut metabolites, the latter may also act on AD significantly. Thomas et al. showed that indole could significantly reduce the expression of INOS, IL-1ß, NLRP3, and other pro-inflammatory genes in BV-2 cells induced by LPS, and also reduce their corresponding protein levels [141]. Intestinal microbiota-derived indoles upregulated the production of aryl hydrocarbon receptor, inhibited the activation of the NF-κB signaling pathway as well as the formation of the NLRP3 inflammasome, and reduced the release of inflammatory cytokines, including TNF-α, IL-6, IL-1β, and IL-18, thus alleviating the inflammatory response of APP/PS1 mice. At the same time, indole treatment also inhibited Aβ accumulation and Tau hyperphosphorylation by affecting tryptophan metabolism and restoring synaptic plasticity, thereby promoting the cognitive and behavioral functions of APP/PS1 mice [142]. Cleavage of tau by caspase-3 at the C-terminal Asp421site was found to be associated with increased aggregation of tau filaments, detectable in both AD transgenic mouse models and the brains of AD patients [143]. TUDCA suppresses apoptosis and reduced caspase-3 activation, thus abolishing caspase-3 cleavage of tau into a toxic species [144]. The level of Aβ42 in the brain tissues of APP/PS1 mice was significantly increased, which could be ameliorated by treatment with the probiotic Clostridium butyricum [105]. Colombo et al. found that SCFA treatment increased amyloid burden in APPPS1 mice [145], whereas Diana et al. treated APP/PS1 mice with SCFAs and found no effect on the amyloid burden [146]. However, the management mode and type of SCFAs used in these experimental studies were different. This may be the reason for the different results, but the specific mechanism of action still needs to be proved through further research.

TMAO levels in the cerebrospinal fluid (CSF) are higher in patients with MCI and AD dementia than in individuals with normal cognitive function, whereby elevated CSF TMAO levels are associated with pathological biomarkers of AD and neuronal degeneration [147]. In a recent study, Zhuang et al. performed a bidirectional MR analysis to assess the potential causative link between intestine flora-dependent metabolites, such as trimethylamine oxide, carnitine, choline, or betaine, and AD risk. They found that TMAO and its precursors did not play a causal role in the development of AD[148]. However, these studies often produced inconsistent results, which may be related to different samples and research methods. Therefore, the specific connection between AD and TMAO as well as the underlying mechanisms also need further investigation in the future.

#### 4.1.2 Parkinson’s Disease (PD)

PD is the second most common neurodegenerative disorder, affecting between 2and 3 % of the population aged 65 or older [149]. It is mainly characterized by motor disorders such as tremors, motor retardation, muscle stiffness, and gait impairment [150, 151]. The neuropathological features of PD include neuronal loss in specific regions of the substantia nigra and extensive intracellular accumulation ofα-synuclein [152].

An important link between microglia and PD has been found in some studies, whereby microglia have a significant neuroprotective role, while impaired and over-activated microglial phenotypes were found to be more abundant in the brains of PD patients [153]. Single-cell sequencing of the human midbrain revealed that microglia in the substantia nigra of idiopathic PD patients were amoeboid, indicating an activated state [154]. PELI1, an E3 ubiquitin ligase highly expressed in activated microglia after treatment withα-synuclein fibrils, induced lysosome breakdown and inhibited autophagy, which promoted α-synuclein transfer via exosomes [155]. Secretion of TNF-α by microglia induces oligomerization of αsyn, which may be part of a vicious pathogenic cycle that further activates microglia. TNF-α induces neuronal death through excitotoxic glutamate-mediated activation of caspases 8 and 10, as well as by interacting interaction with the TNF receptor [156]. Immunoglobulins derived from PD patients activated Fcγ receptor positive microglial cells in mice, which further promoted the cell death of dopaminergic neurons [157].

There is increasing evidence that intestinal metabolites have an effect on microglia, implying that they may also have some connection with PD. Flavanol preparations containing phenolic acid metabolites of gut microbiota are effective in modulating the development and progression of motor dysfunction in mouse and Drosophila models of PD, where they appear to interfere with α-synuclein misfolding or the resulting inflammation [158]. PD patients have lower fecal concentrations of butyric, propionic, and acetic acid which is accompanied by higher serum levels of acetic acid and propionic acid. Except for propionic acid, fecal SCFAs were negatively correlated with disease severity, while plasma acetic acid, propionic acid, and valerate were positively correlated [5]. PD patients also exhibit an altered composition of the gut microbiome, with an increase in pro-inflammatory Enterobacteriaceae and a decrease in bacteria that protect against autoimmune and inflammatory processes including butyrate-producing bacteria [159]. The levels of intestinal metabolites produced by these bacteria are also correspondingly reduced, which may be related to the pathogenesis of PD. Based on the metabolic changes observed in the appendix of PD patients, it can be inferred that the secondary bile acids lithocholic acid (LCA)and deoxycholic acid (DCA) were increased in the ileum of PD patients. DCA and LCA are highly hydrophobic, whose rise can produce proinflammatory effects as well as direct cytotoxicity. These effects may drive the accumulation of pathological α-synuclein aggregates that are transmitted from the gut to the brain via retrograde transport, leading to the development of PD [160, 161]. In another study, researchers found that the ratio of pro-inflammatory TMAO to the putative anti-inflammatory metabolite butyric acid was significantly higher in PD patients than in controls, indicating a pro-inflammatory shift in the metabolite profile of PD patients [162]. However, two recent studies on TMAO obtained different results, one demonstrating an increase in plasma TMAO in PD patients, the other a decrease [163, 164]. The heterogeneity of the PD population (course of the disease, lifestyle, treatment status) may explain the discrepancies between these studies. Further studies are needed to confirm the specific relationship between TMAO and PD as well as the possible mechanism underlying this link.

### 4.2. Neurodevelopmental disorders.

#### 4.2.1. Autism spectrum disorder (ASD)

ASD is a neurodevelopmental disorder caused by abnormalities in different parts of the brain. Individuals diagnosed with ASD exhibit deficits in social interaction and communication (developing, maintaining relationships, and understanding social cues) as well as repetitive/stereotypical behaviors that vary in severity combined with hypersensitivity to environmental sensory signals [165, 166].

Microglia play a significant role in brain development by regulating inhibitory and excitatory pathways, promoting synaptic plasticity, neuronal migration, neurogenesis, and immune regulation [167], and are also considered to play a possible role in the pathogenesis of ASD. Previous studies have found that altered microglial density and morphology, altered motility patterns, as well as differentiated chromatin accessibility and transcription profiles contribute to the development of ASD [167]. However, it remains to be determined whether microglia play a pathogenic role in the occurrence or manifestation of ASD, or if microglial activation is a secondary effect of abnormal brain development. Xu et al. believe that deficiencies in negative translation regulator genes are sufficient to change the function of microglia to drive ASD. In addition to an increased phagocytosis capacity, these microglia exhibited reduced mobility and impaired synaptic pruning, ultimately leading to higher synaptic density and higher excitatory neurotransmission compared to wild-type mice, ultimately driving the development of ASD-like behavior [168]. Since both activated and resting microglia secrete neurotrophic factors, neurotoxic factors, cytokines, and other soluble factors associated with ASD, microglia may use these mediators to influence multiple neuronal functions and form synaptic connections [169, 170].

The link between intestinal metabolites and microglia may also be associated with ASD. In one study, the authors found that patients with ASD had altered levels of SCFAs and volatile organic compounds, including indole and other tryptophan metabolites [171]. In previous studies, SCFAs were found to regulate synaptic activity through the BBB and VN, influencing ASD-like behavior [172, 173]. In a valproate-induced animal model autism, the researchers found that the content of SCFAs in ASD animals was decreased and intestinal tissue disintegration was observed, accompanied by increased levels of the pro-inflammatory factor TNF-α, while the abundance of microglia was increased. At the same time, the experimental results indicate that acetyl-L-carnitine can increase the content of acetic acid and butyric acid in animals and achieve the effect of ameliorating behavioral disorders [174]. According to research, more than 25% of children with ASD have elevated levels of serotonin in their blood, suggesting a link between serotonin and ASD[175]. Recent studies have also confirmed that microbial metabolites specifically evoke the core behavioral symptoms of ASD in mice [176]. Compounds produced by pathogenic bacteria, including lipopolysaccharides and opioid peptides, enter the bloodstream, causing inflammation and damaging nerve tissue, as well as compromising the integrity of the intestinal barrier. These dangerous metabolites also disrupt neurotransmitter function in the brain, leading to abnormal behavioral patterns such as reduced social interaction and inappropriate language [177]. Endocannabinoids are bioactive lipids, produced from arachidonic acid. It has been proved that the signal strength of endocannabinoids is positively correlated with the degree of mood disorders, and their involvement in mood and neurodevelopmental disorders may be related to oxytocin signaling, while also causing changes in hippocampal neuron-glia-microglia communication [178]. However, the effects of antibiotic therapy or personalized diet on patients with ASD in some of the studies may have affected the results, and more reliable data are needed to explain the underlying mechanisms.

#### 4.2.2. Attention deficit hyperactivity disorder (ADHD)

ADHD, a quite common neurodevelopmental disorder, is defined by impairment symptoms such as inattention, impulsivity, and hyperactivity [179]. ADHD has been linked to an increased risk of a range of other mental health problems, including depression, anxiety, oppositional defiant disorder, antisocial behavior, and substance abuse [180].

The etiology of ADHD is complex, and its specific mechanisms are still unclear. The spontaneously hypertensive rat (SHR) model showed similar behaviors to those of ADHD patients, including increased motor activity, impulsivity, and inattention [181]. Immuno-fluorescence staining was used to detect the activation of CA1 in the hippocampus and microglia in the prefrontal cortex. Compared with normal rats, the expression of CA1 in the hippocampus and microglia in the prefrontal cortex of the SHRs was significantly increased [182]. This suggests that microglia may be involved in the pathogenesis of ADHD.

A study has shown that treating pups with monosodium glutamate leads to reduced emotional behavior and aggressive behavior, later in life, which can be abrogated by vagotomy at the subphrenic level [183]. This observation implies that ADHD is associated with VN. It has been mentioned above that many intestinal metabolites act on microglia through the VN, which may be one of the ways that ADHD is associated with intestinal metabolites. Through metabolomics analysis, it was found that intestinal metabolites were indeed associated with ADHD [180]. Dopamine and norepinephrine, which can be produced by gut microbes, are involved in the pathophysiology of ADHD and play vital roles in behavioral, cognitive, and emotional functions [184]. Furthermore, researchers found that ADHD patients had increased levels of bifidobacteria, which produce an enzyme involved in the synthesis of phenylalanine, a precursor to dopamine [185]. One clinical study showed that ADHD patients had lower circulating serotonin concentrations, which were also observed in GF mice, and that oral tryptophan alleviated ADHD symptoms [186]. In recent studies, it was found that the traditional Chinese concoction Dimu Ningshen has a good therapeutic effect on ADHD, which may be based on its effect on intestinal flora and affecting corresponding circulating metabolites [187]. In the future, the regulation of metabolite composition by altering the composition of intestinal flora may become an important means to treat neurodevelopmental disorders.

## 5. Discussion

As a research hotspot in recent years, the link between the intestine and brain diseases has been widely discussed. As innate immune cells in the CNS, the influence and effect of microglia in neurodegenerative diseases have been proved in many studies. In addition, the function of microglia is also closely related to the gut-brain axis. The integrity and functional status of the important structures in the gut-brain axis, such as the GBB, the BBB, and the VN, have certain effects on the activity of microglia. Depending on these important structures in the gut-brain axis, intestinal metabolites are also directly or indirectly involved in microglial regulation. However, the current studies of microglial interactions with gut metabolites may be just the tip of the iceberg. The emergence of new tools, such as single-cell transcriptomics (scRNA-seq), has greatly improved our ability to characterize cellular heterogeneity, allowing us to deepen our understanding of the relationship between microglia and intestinal metabolites [188]. Recently, researchers have established a single-cell transcriptome analysis method called Live-seq, which maintains cell viability during RNA extraction using flow cytometry microscopy, thus allowing the coupling of a cell's ground-state transcriptome to its downstream molecular or phenotypic behavior [189]. This technique allows the removal of other possible cellular influences and allows a more detailed understanding of the mechanisms underlying the interaction between gut metabolites and microglia. At the same time, the development of advanced microscopy techniques, such as two-photon microscopy, and multi-photon microscopy, allows us to observe the activity of microglia in the living brain [190, 191]. Using these new research techniques, the development of drugs targeting microglia and intestinal metabolites will be further developed. Previous studies have shown that probiotics can affect anxiety by modulating the levels of neurotransmitters, as well as reducing cortisol production, directly affecting stress, anxiety, and depression-related behaviors [192, 193]. A large amount of clinical and preclinical evidence also indicates that probiotics can affect the occurrence or progression of Parkinson's disease by affecting the composition of the intestinal flora, enhancing intestinal epithelial integrity, and reducing the pro-inflammatory response [194]. In the current market, there are also many probiotics that show promising results in the treatment of neurological diseases. Pterostilbene, a famous natural stilbene derivative, is abundant in berries, nuts, and other plant foods [195, 196]. It has been shown that pterostilbene alleviates neuroinflammation in mice with diabetic cognitive impairment by inhibiting oxidative and carbonyl stress injury, thus preventing the activation of microglia and loss of dopaminergic neurons. Further studies have shown that Astragalus can reduce the levels of lipopolysaccharide, regulate the TLR4/NF-κB signaling pathway in the colon and brain, reduce the release of inflammatory factors, improve the homeostasis of intestinal microbiota, increase the level of short-chain fatty acids as well as their receptors, and inhibit the loss of intestinal tight junction proteins [197]. These studies provide a new basis for dietary intervention in the treatment of neuroinflammation. Shugan granules can alleviate the neurofibrillary tangles and neuronal degeneration induced by chronic restraint stress, while also promoting the recovery of the intestinal barrier following injury. SGKL increases the phosphorylation of the PI3K/Akt/mTOR pathway in microglia by altering intestinal flora and metabolites, thus inhibiting the LPS-induced activation of microglia to improve the hippocampal behavior and inflammatory response [198]. The analysis of intestinal flora and metabolites showed that spinal cord injury caused an imbalance of intestinal flora and decreased butyrate levels, while resveratrol restored the balance of intestinal flora, increased the concentration of corresponding metabolites, and promoted functional recovery after spinal cord injury by inhibiting the activation of spinal microglia [199]. With high levels of butyric acid in the brain, rifaximin increases the relative abundance of Ruminococcaceae and Lachnospiraceae, increases the content of anti-inflammatory factors released by microglia and prevents neurogenic abnormalities caused by chronic unpredictable mild stress [200]. Intestinal metabolomics is a complex field, and the effects of metabolites on microglia are affected by many factors, such as dose, environment, and physiological state, among others. In many cases, there is no single metabolite that plays a decisive role, and the specific mechanisms of action need to be further clarified. Further elucidating and studying these mechanisms will enable us to better understand the role of intestinal metabolites on microglia and discover potential therapeutic measures. In the future, the regulation of intestinal metabolites can be used as an important target to affect the phenotype and function of microglia, so as to prevent and treat neuroinflammation and neurodevelopmental disorders, as well as to reduce the effects of cognitive impairment and memory decline affecting millions of patients around the globe.

## 6. Conclusions

Intestinal metabolites, produced by the interplay between the host’s intestinal enzymes and those of intestinal microbes are an indispensable part of the gut-brain axis. As such, they either indirectly act on microglia by changing the structure of the gut-brain axis, or directly regulate microglia. Intestinal metabolites play a role in shaping the nervous system by affecting the pathways of microglia and the levels of inflammatory and neurotrophic factors, which are considered to be the regulated targets of neurological and psychiatric diseases. Further understanding of the role of gut metabolites in the regulation of microglia and the gut-brain axis will allow researchers to better understand the underlying mechanisms and provide a new way to intervene in the expression and status of microglia. Novel therapeutic interventions based on targeting intestinal metabolites will provide a new direction for the pharmacological treatment of neuropsychiatric diseases and may become a mainstream direction for the treatment of related disorders in the future.
